# Role of Glycoproteins during Fruit Ripening and Seed Development

**DOI:** 10.3390/cells10082095

**Published:** 2021-08-15

**Authors:** Angela Mendez-Yañez, Patricio Ramos, Luis Morales-Quintana

**Affiliations:** 1 Multidisciplinary Agroindustry Research Laboratory, Talca 3467987, Chile; amendez.ya@gmail.com; 2 Centro de Investigación de Estudios Avanzados del Maule (CIEAM), Universidad Católica del Maule, Talca 3460000, Chile; pramos@ucm.cl; 3 Multidisciplinary Agroindustry Research Laboratory, Instituto de Ciencias Biomédicas, Facultad de Ciencias de la Salud, Universidad Autónoma de Chile, Talca 3467987, Chile

**Keywords:** fruit ripening, glycoproteins, *N*-glycosylation, seed development, quality fruit

## Abstract

Approximately thirty percent of the proteins synthesized in animal or plant cells travel through the secretory pathway. Seventy to eighty percent of those proteins are glycosylated. Thus, glycosylation is an important protein modification that is related to many cellular processes, such as differentiation, recognition, development, signal transduction, and immune response. Additionally, glycosylation affects protein folding, solubility, stability, biogenesis, and activity. Specifically, in plants, glycosylation has recently been related to the fruit ripening process. This review aims to provide valuable information and discuss the available literature focused on three principal topics: (I) glycosylations as a key posttranslational modification in development in plants, (II) experimental and bioinformatics tools to analyze glycosylations, and (III) a literature review related to glycosylations in fruit ripening. Based on these three topics, we propose that it is necessary to increase the number of studies related to posttranslational modifications, specifically protein glycosylation because the specific role of glycosylation in the posttranslational process and how this process affects normal fruit development and ripening remain unclear to date.

## 1. Introduction

Glycosylation is the process of adding carbohydrates to a protein and is an essential and important process to produce protein posttranslational modification (PTMs) in eukaryotic cells [1]. Glycosylation is involved in many important cell life processes, such as differentiation, recognition, development, signal transduction, and immune response; additionally, glycosylation affects protein folding, solubility, stability, biogenesis, and activity [2,3]. Moreover, in plants, glycosylation has been related to the fruit ripening process [4,5,6].

With respect to the biochemical process, glycosylation involves the covalent linkage of a sugar moiety or glycan to a mature protein in the endoplasmic reticulum (ER) and the Golgi apparatus [7]. The biosynthetic pathway of these sugar monomers involved in the glycosylation process overlaps with the biosynthesis of ascorbate as well as with those used for cell wall polysaccharide precursors, including mannose, fucose, xylose, and galactose [3].

The basic molecular machinery participating in protein glycosylation differs slightly between species in which these PTMs occur based on the specificities of some enzyme transferases and hydrolases on the faces of the Golgi in different species. Thus, specific glycosylation events are generated based on residues and particular bonds in each kingdom. Similarly, glycosylation events could change in time and space, depending on biotic or abiotic stress, external conditions, and the genetic background of the plant.

The intrinsic effect of glycosylation in different organisms has been studied. However, current knowledge of complex glycans in plants is very limited. Thus, this review critically organizes information from recent years about glycan-specific functions in various biological processes related to fruit development and ripening.

## 2. Different Glycosylation Types

There are three different types of glycosylation: *C*-glycosylations, *N*-glycosylation, and *O*-glycosylation. These types of glycosylation events are distinguished based on the location where the carbohydrate is attached to the amino acid residue of the protein [8]. *C*-glycosylation occurs when the point of glycosylation is the carboxyl group of the tryptophan residue, *N*-glycosylation occurs when the point of glycosylation is the amino group of the side chain of the asparagine residue, and *O*-glycosylation occurs when the point is the hydroxyl group of the side chains (2*S*,4*R*)-4-hydroxyproline (Hyp) [9].

### 2.1. N-Glycosylations

*N*-glycosylations are as posttranslational modification processed in the endoplasmic reticulum and Golgi apparatus [7]. Currently, numerous studies have described *N*-glycosylations in plants, including the synthesis and molecular machinery involved [10,11]. In all eukaryotes, glycosylation involves a lipid-linked oligosaccharide and a glycan block that binds to asparagine (N) followed by any residue (X) and then serine or threonine (S/T) (the sequence is termed NXS/T) and is the substrate of the enzyme oligosaccharyltransferase [12]. Modifications realized by glycosyltransferases and hydrolases enzymes in different Golgi faces produce an *N*-glycosylation event that promotes a particular biological role.

In contrast to mammals, insects, or yeasts, glycosylation events in plants involve specific glycans, such as xylose and fucose [13]. For example, β1,2-xylosyltransferase and α1,3-fucosyltransferase enzymes. Mutation or silencing of genes that code for the two enzymes mentioned above to produce viable plants and phenotypes under laboratory conditions [14]. Nagashima et al. [3] showed that the functions of *N*-glycosylation events in plants are related to the protein folding, protein receptors, biogenesis, cell wall biosynthesis, and salt stress tolerance. A summary of the main evidence related to *N*-glycosylations in plants is shown in Table 1. Additionally, it has been recently found that *N*-glycosylations vary in space and time. Thus, the proportional variability of the *N*-glycans and subcellular distribution has been verified [15].

#### 2.1.1. *N*-Glycosylation and Plant Development

**Figure 1:**
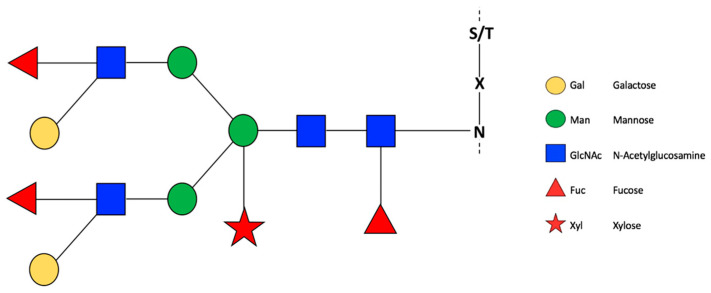
General representation of *N*-glycosylation in plants. Glycans are represented according to Consortium for Functional Glycomics (CFG). Recovered with modifications of Wang et al. [32].

Reports of *N*-glycosylated enzymes, related to fruit ripening and the shelf life of fruit are scarce. Nevertheless, efforts have been directed to understanding the role of *N*-glycosylation in plant development (Figure 1). For example, mutants related to photosynthesis from *Arabidopsis thaliana* enzymes α1,3-fucosyltransferase (alg3-3*)* and *N*-acetyl glucosaminyltransferase I (cgl1-1), which have defects in Dol-PP-linked glycans and an absence of complex glycans, respectively, were studied [23]. In these mutants, a decrease in the capacity to capture and transfer excitation energy was observed by increasing light levels, a decrease in dry biomass, and a decrease in photosynthetic capacity and functional decomposition of chloroplast-located protein [23]. In calcium signal mutants of *A. thaliana*, such as cce2 and cc3, a decrease in the enzymatic function of proteins encoded by the alleles cce2/cc3 was observed, where ALG3, which codes for the α-1,3-mannosyltransferase enzyme, is responsible for the assembly of the first glycan Glc3Man9GlcNAc in the endoplasmic reticulum. Glycosylation defects compromise the immune response of plants, altering the molecular patterns related to defense against microorganism responses, and defects in glycosylation can affect microorganism virulence [29]. In this sense, plant organisms present molecular patterns associated with pathogens (MAMPs/PAMPs), which activate a series of molecular events that are triggered when producing an infection or damage; these patterns of recognition can be lipopolysaccharide, peptidoglycan, extracellular proteins, extracellular ATP or polygalacturonates [29]. In the case of MAMP signaling, plants with low levels of recognition patterns can be susceptible to pathogens, as in the case of tomatoes, such as *Pseudomonas syringae*. As described by Lin et al. [30], infection occurs through the interaction of the host cell surface proteins and the pathogen, where a large portion of these extracellular proteins is glycosylated [30]. This mechanism explains how glycosylation in plants can act as a barrier against pathogens or induce resistance [30].

Mutations in key enzymes of the *N*-glycosylation pathway are viable in some plant species but lethal in other plant species. The reason for this difference remains unknown [31]. However, when investigating the molecular responses of the plant, internal (as the genotype) and external (as the environment) events that could indirectly affect the expression of certain genes must be considered.

#### 2.1.2. *N*-Glycosylation and Fruit Ripening

Different efforts have been made to understand the relationship between *N*-glycosylation and fruit ripening. In this regard, Ghosh et al. [33] evidenced that silencing through RNA interference in α-mannosidase (α-Man) and β-d-*N*-acetylhexosaminidase (β-Hex), two *N*-glycoprotein modifying enzymes present in *Capsicum annuum* fruits, delayed fruit deterioration until seven days post-harvest, providing fruits up to twice as firm as the control. The relationship of the β-Hex enzyme in ripening fruit was determined in *Fragaria x ananassa* (commercial strawberry). Transcript levels of two genes that code for two members of this enzyme family, increase during ripening, and the inhibition of its enzymatic activity with alginate oligosaccharides allow us to extend the shelf life of the fruit [26] (Table 1).

Similar results were obtained by Meli et al. [34], who showed that α-Man and β-Hex suppression in *Solanum lycopersycum* produced an increase in firmness (2.5 fold greater than that of the control) and consequently prolonged the shelf life by 30 days.

Varki [35], described a general classification of the biological role of glycans: (1) Structural and modulatory roles: Glycoproteins help the cell perceive and interact with the surrounding microenvironment. Polysaccharides, for example, are used to organize the membrane and the extracellular matrix and provide structural support, adhesion, protection, and elasticity to the cell. Specifically, the cell wall changes dynamically based on the action of enzymes with catalytic activity in cells, different sugars in the cell wall, and glycans in proteins. Similarly, in some cases, glycosylation affects the diffusion and solubility of macromolecules and provides a defense against pathogens and proteases. (2) Intrinsic recognition: Glycoproteins facilitate internal communication, triggering cascades of signals or reactions in response to processes that occur inside the cell. For example, internal recognition of some internal signals triggers endocytosis or phagocytosis. (3) Extrinsic recognition: Interaction with pathogens and symbionts. Glycans participate mainly in interaction and recognition. Specifically, microorganisms recognize glycans, and the host organism simultaneously produces glycans as defense mechanisms. On the other hand, the adhesins produced by microorganisms facilitate their ability to bind to the host cell. Regarding viruses, glycoproteins such as hemagglutinins, allow viruses to achieve success in infection by agglutinating erythrocytes. (4) Molecular mimicry of host glycans: Evolution has allowed pathogens and hosts to change in such a way that the pathogen can mimic some of its molecular mechanisms, such as host-specific glycosylation events and some host cell-specific molecular patterns (SAMPs). On the other hand, some microorganisms are capable of capturing free glycans and adding them to their proteins to mask them within the host cell. Within each category, a list of specific biological roles related to specific glycosylation events has been reported.

**Figure 2:**
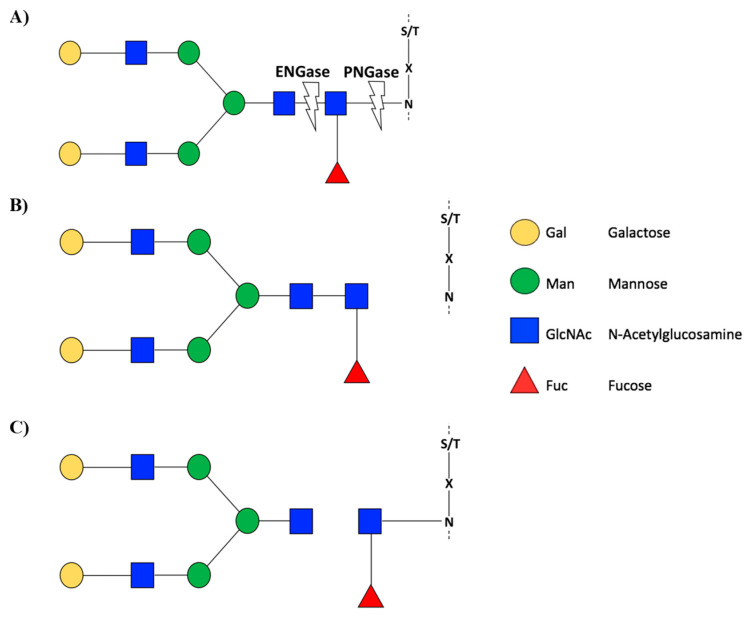
Schematic representation of the EnGase and PNGase enzyme activity. (**A**) PNGase F has activity in the glycosidic bond between asparagine amino acid and GlcNAc glycan, while, ENGase has activity in the glycosidic bond between two GlcNAc glycans. As result, with PNGase F glycosylation is completely cleaved (showed in **B**). ENGase leaves a GlcNAc attached to the asparagine amino acid (showed in **C**). Glycans are represented according to Consortium for Functional Glycomics (CFG). Recovered with modifications of Karav et al. [38] and Fairbanks [39].

Recently, it has been reported that the *N*-glycoproteome of a plant changes in space and time. Zhang et al. [36] described the glycoproteome of two contrasting fruit stages (ripe stage contrasting with green stage) of *Solanum lycopersicum*. Comparison of the *N*-glycoproteome in both stages showed 553 *N*-glycosites and 363 *N*-glycoproteins. Of these, 252 *N*-glycosites and 191 *N*-glycoproteins were differentially expressed between the two stages. Similarly, the *N*-glycoproteome has been used as a powerful technique to analyze *N*-glycoproteins and *N*-glycosites in tomato fruits under saline stress conditions, which led to a decrease in *N*-glycosites. This evidence indicates that *N*-glycosylations are reprogrammed under stress conditions. For example, under salt stress conditions, glycoproteins could alter their folding and/or change the activity level [36]. The loss of glycans in proteins can be mediated by enzymes that cleave the glycosidic bonds in the branches of glycans, such as *N*^4^ (*N*-acetyl-glucosaminyl) asparagine amidase (PNGase) and endo *N*-acetyl-beta-D-glucosaminidase (ENGase) (Figure 2). In young and developing tissues, such as apical buds, flowers, and leaf blades, PNGase and ENGase enzymes are mostly active but exhibit different levels of gene expression. In the fruit ripening stage in *S. lycopersicum* fruits, the transcript levels of these enzymes together with other proteases are significantly decreased [37].

In addition to studying enzymes with deglycosylase activity, such as α-Man and β-Hex, other enzymes that are related to plant cell wall metabolism have been investigated during the fruit ripening process. Méndez-Yáñez et al. [5] biochemically characterized the FcXTH1 enzyme (Table 1). This enzyme is related to the fruit ripening process, specifically with cell wall remodeling and fruit softening in different fruits, including Chilean strawberry [5]. To describe the effect of glycosylation on enzyme activity, the authors used bioinformatics tools, such as protein modeling, in silico protein-ligand interaction analysis by molecular docking and molecular dynamics simulations. All these studies were validated by kinetic and biochemical assays. The principal result was that glycosylated proteins are more stable than non-glycosylated proteins [5]. In addition, this finding is not the only example of the positive effect of glycosylation on XTH activity; the same enzyme is expressed in heterologous systems and exhibits changes in biochemical parameters and enzyme stability [5]. For example, in PttXET16A, an XTH of *Populus tremula x tremuloides*, site-directed mutagenesis studies were performed (substitution of serine for asparagine in the glycosylation site), and the mutation did not produce a significant reduction in XET activity [25]. Additionally, the removal of *N*-glycan by endoglycosidase H (Endo H) treatment did not significantly modify the XET activity in PttXET16A [25]. In contrast, the activity levels of AtXTH22 and AtXTH24, two XTH enzymes identified in *A. thaliana*, were significantly reduced by PNGase F treatment [40]. Similar results showed the stability of the HvXET6 enzyme during storage at 4 °C. The analysis revealed that HvXET6 treated with PNGase F gradually lost its activity, with a decline of approximately 20–25% reduction in activity after 6 days. However, when HvXET6 was treated with Endo H, the enzyme remained almost fully active even after 6 days of storage [41]. Interestingly, Endo H cleaves the chitobiose core of high mannose *N*-linked glycoproteins, whereas PNGase F releases almost all types of *N*-linked glycans between the innermost GlcNAc and asparaginyl residues [41]. The authors indicate that the HvXET6 protein requires only one GlcNAc to maintain XET activity [41]. However, this affirmation is not completely true because the kinetic parameters (K_M_ and k_cat_) of the HvXET6 enzyme treated with PNGase F (after 16 h of incubation at 20 °C) differ from those of glycosylated HvXET6^2^: the kcat/KM ratio was increased by approximately 40% [41]. It is thus possible that *N*-glycans have a differential role in XET activity, and this apparent crucial role is more important for some XTHs than others. However, a clear understanding of the reasons for this difference is lacking.

Recently, reported research on complex *N*-glycans has focused on *N*-glycosylation enzyme pathway silencing-mediated RNAi. Specifically, a reduction in *N*-acetyl-glucosaminyltransferase I (GNTI) activity produces necrotic stems, early fruit abscission, incomplete ripening, and susceptibility to infections [31]. In plants treated with α-mannosidase II (MANII) RNAi, the fruits showed a normal appearance. However, the fruits did not contain seeds or only a few enlarged seeds, and rolled leaves were observed in the reproductive stage [31].

### 2.2. O-Glycoproteins in Fruits and a World Not Yet Explored

**Figure 3:**
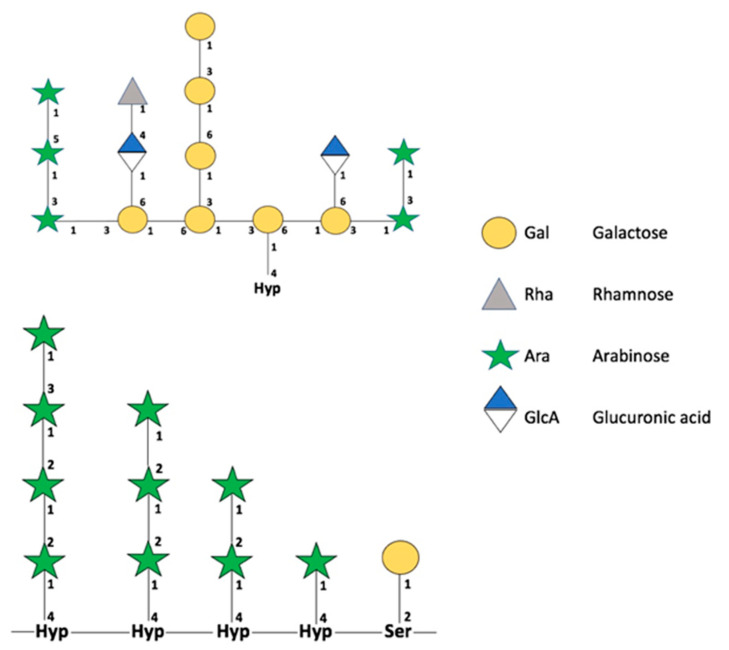
Schematic representation of *O*-glycans. The upper image summarizes the possible structures found in *O*-glycans in type II AGPs. The bottom image summarizes the possible *O*-glycosylations in plant EXTs. Representations of monosaccharides are based on the official notation of the Consortium for Functional Glycomics (CFG). Retrieved with modifications from Tan et al. [45] and Nguema-Ona et al. [46].

The first report describing *O*-glycosylations was published by Torres et al. [42]. *O*-glycosylations are less studied than *N*-glycosylations, and many processes and studies related to their mechanism of action have not yet been described. The cellular pathways involved in *O*-glycosylation are unclear; however, some studies observed the importance of repeated proline (Pro) residues in the different glycans. Although not all the components of the pathway are clear, we currently know that *O*-glycosylation differs in mammals and plants. Specifically, the first hydroxylation of oxygen occurs in a Ser or Thr residue in mammals, versus a Pro residue in plants [43]. The change in Pro to hydroxyproline (Hyp) (Figure 3) is catalyzed by proline hydroxylase enzymes (P4Hs). The group of hydroxyproline-rich glycoproteins in plants is formed by: extensins (EXTs), arabinogalactan proteins (AGPs), and proline-rich proteins (PRPs) [43]. Pro hydroxylation produces a change in the molecular charge of the microenvironment and generates a conformational change and interaction between proteins to produce hydroxyl groups, allowing posttranslational modifications. Thus, Hyp is a signal that the molecular machinery recognizes as a flag, indicating the site where *O*-glycosylation must be performed.

#### Reports of *O*-Glycosylations in Plants

In *A. thaliana*, it has been demonstrated that *O*-glycosylation, specifically arabinosylation in EXTs, is essential to root hair elongation. Mutant lines deficient in the P4H gene exhibited a lower root hair size compared with controls [44]. Recently, the first large-scale *O*-glycosylation study in plants was reported, where Xu et al. [27] found 262 *O*-glycosylated proteins with an important role in *A. thaliana*. However, the role of *O*-glycosylation in fruit ripening has not yet been determined.

## 3. Experimental Techniques to Identify *N*- and *O*-Glycosylations

Ruiz-May et al. [47] reviewed analytical and classical technologies to identify and characterize *N*-glycosylations in plants. Since then, many publications have used these and other novel technologies to study the *N*-glycoproteome in plants. For example, mass spectrometry (MS) remains the primary experimental technique used to characterize *N*-glycosylations in eukaryotes. The technique typically used to recognize the glycosylation events present in a protein requires deglycosylation, which leads to the loss of information. This process makes it impossible to know the specific point to which a certain glycosylation was attached. Therefore, the polypeptide fragmentation technique, which involves hydrolysis and subsequent analysis of the glycosylated sites and their respective glycans, remains an excellent alternative [48]. Stravenhagen et al. [49] proposed a workflow for the site-specific analysis of an *N*-glycosylation or *O*-glycosylation site. Additionally, a consensus sequence is necessary for the identification of potentially *N*-glycosylated sites, but the occupation of an *N*-glycosylation site is not mandatory [50].

The current experimental methods for *O*-glycan characterization and separation have been summarized in the work of Wilkinson and Saldova [51], where they describe the main glycan release methods: enzymatic, reductive β-elimination, nonreductive by β-elimination (hydrazinolysis and thousand alkalines), and oxidative release of natural glycans. On the other hand, the main methods for the separation and analysis of glycans include high-performance liquid chromatography (HPLC); UPLC, or UHPLC (Ultra High-Performance Liquid Chromatography); exoglycosidase arrays; mass spectrometry (MS); MS ion fragmentation; capillary electrophoresis (CE); nuclear magnetic resonance (NMR) spectroscopy; and lectin affinity.

Mewono et al. [52] performed a review of three chemical inhibitors of the enzymatic activity of prolyl 4-hydroxylase (P4H): (1) 3,4-dehydro-l-proline, an analog of Pro that is capable of being incorporated into the polypeptide chain; (2) ethyl 3,4-dehydroxy benzoate, which binds to the active site of the P4H enzyme, and (3) α-α-dipyridyl, a chelator of the key P4H cofactor. This technique could be useful for studying *O*-glycosylated proteins.

## 4. Bioinformatics Glycosylations Tools in Plants

A large number of online applications and databases are available to perform glycoinformatics. Muthu et al. [53], suggested a classification of databases with bioinformatics resources grouped in three categories: (1) proteins; (2) enzymes and pathways to build glycans, and (3) carbohydrate structural databases. The authors provide a list of tools and online resources to study glycosylations. For molecular modeling of glycosylated proteins, Frank and Schloissnig [54] provided a pipeline and valuable details to the design of a protein with glycosylation. A force field used for molecular modeling in an environment with carbohydrates has been developed and detailed by Park et al. [55].

Combining experimental data with in silico analysis [56] is a useful technique that can potentially reduce research times required to model a glycoprotein and perform subsequent minimization and molecular dynamics analyses. Research published based on this type of combined technique, increases confidence in dry-lab technologies, reducing experimental search times.

Specifically, in plants, different resources currently exist for glycosylations studies. Johnson et al. [57] developed a pipeline to identify HRGPs in plants to facilitate focused efforts on the search and subsequent study of *O*-glycosylated proteins. Another important resource available online is Plant PTM Viewer (https://www.psb.ugent.be/webtools/ptm-viewer/, accessed on 4 August 2021) [58]. Using this resource, a sequence can be analyzed, and the putative sites of posttranslational modifications, including glycosylations, can be predicted. The database is supported by experimental data, and information included in this database has been deposited in other databases, such as UniProt, PubMed, and InterPro. The main recommendation when analyzing posttranslational modifications is to use different databases and applications in conjunction with the literature and previous experimental results, if available. Although algorithms and results obtained and generated by machines are quite reliable, it is necessary to remember that the biological interpretation has not yet been fully elucidated.

Phyton scripts are also very useful for glycosylation studies. For example, Glycosylator is a phyton framework for the investigation of glycoproteins using the CHARMM force field [59]. However, for novices in the field of informatics, various applications, programs, and web servers with an easy-to-use graphical interface are available on the internet.

**Figure 4:**
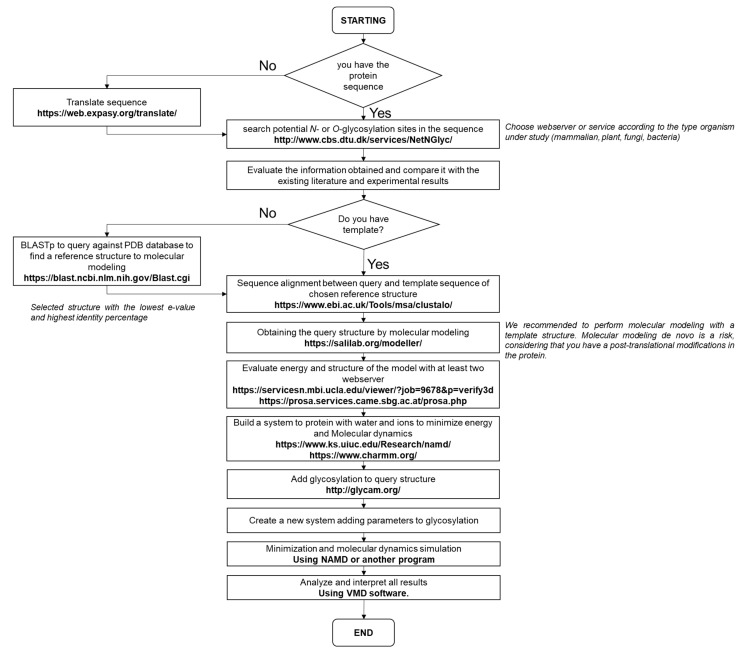
The pipeline proposal is based on the comparative modeling methodology. The pipeline was designed according to the methodology described by Méndez-Yáñez et al. [5].

To study glycosylated proteins in silico (Figure 4), we proposed the following steps: (1) All the biological and experimental information that is based on the enzyme or protein under study must be considered; (2) Evaluate or predict its secondary structure; (3) Analyze the putative glycosylation sites and whether these sites could be glycosylated based on their structure; (4) Once the glycosylated sites are defined, build the glycan and the glycosylated protein to be studied, and (5) Choose force fields for molecular dynamics and simulation, that also consider the parameters for glycan structures.

## 5. New Perspectives and Challenges

Although potential activity or gene expression can be evidenced a priori, once the protein has been transcribed and located in the site where it will perform its function within the cell, it is possible to elucidate whether a gene has an impact at biological and biochemical levels. Clearly, information regarding glycosylated proteins, transglycosylase and hydrolase enzymes, glycomes, and other investigations related to fruit ripening and postharvest are lacking. In addition, the importance of studying *O*-glycosylations in relation to fruit ripening has not yet been determined. However, we believe that in the near future, glycomics will be an important area of study, similar to other omics.

The relationship of this type of PTM and fruit ripening could undoubtedly be the key to prolonging the useful life of the fruit or to understanding the essential molecular events for optimal fruits developments.

## Figures and Tables

**Table 1:** Summary of principal research about *N*-glycosylation and *O*-glycosylation in plant proteins since 2000. This table contains glycosylated proteins, glycosyltransferases, glycosylhydrolases, and transglycosylase.

Enzyme or Protein Type	Organism	Enzyme	Function	Reference
Glycosylated enzymes and proteins	*Arabidopsis thaliana*	AtERO1	Identification of *N*-glycosylation sites of ER oxidoreductin-1.	[16]
	*Arabidopsis thaliana*	AtERO2	Identification of *N*-glycosylation sites of ER oxidoreductin-2.	[16]
	*Nicotiana benthamiana*	Cannabichromenic acid synthase	Mutagenesis is a glycosylated enzyme to produce tetrahydrocannabinolic acid in tobacco.	[17]
	*Pyrus bretschneideri*	Fasciclin-like arabinogalactan protein family	Identification and function analysis of this family of proteins.	[18]
Transferase	*Solanum tuberosum* L. *Nicotiana tabacum* L. *Arabidopsis thaliana*	*N*-acetyl glucosaminyltransferase I	Enzyme that begins the complex *N*-linked glycans.	[19]
	*Nicotiana benthamiana*	*N*-acetyl glucosaminyltransferase I	Glycoengineering to produce human enzyme glucocerebrosidase	[20]
	*Nicotiana benthamiana*	α1,3-fucosyltransferase	Inhibition to produce human-like *N*-glycan structure.	[21]
	*Nicotiana benthamiana*	β1,2-xylosyltransferase	Inhibition to produce human-like *N*-glycan structure.	[21]
	*Arabidopsis thaliana*	*O*-fucosyltransferase	*O*-glycosylation regulates transitions in developmental stages.	[22]
	*Arabidopsis thaliana*	cgl1-1	Knockout of *N*-acetyl glucosaminyltransferase I.	[23]
	*Arabidopsis thaliana*	β-glucuronosyltransferase	Identification of two β-glucuronosyltransferase involved in the biosynthesis of mucilage polysaccharides.	[24]
	*Arabidopsis thaliana*	alg3-3	Knockout of α1,3-mannosyltransferase	[23]
Transglycosylase	*Populus tremula x tremuloides*	PtXTH1	Enzyme that modifies the cellulose-xyloglucan network during wood formation.	[25]
	*Fragaria chiloensis*	FcXTH1	Enzyme that modifies the cellulose-xyloglucan network during fruit ripening.	[5]
Hydrolase	*Fragaria x ananassa*	β-Hex	Influence of β-Hex in the strawberry ripening	[26]
*O*-glycosylation	*Arabidopsis thaliana*	*O*-glycosylation screening in plant peptides	Identification of 262 proteins *O*-glycosylated with regulatory functions.	[27]
	*Nicotiana benthamiana*	Human Granulocyte-Colony Stimulating Factor	Transient expression via agroinfiltration	[28]

## Data Availability

Not applicable.
